# The Diet Structure and Body Mass Index Among Polish Preschool Children in Relation To Their Place of Residence

**DOI:** 10.34763/devperiodmed.20182202.153159

**Published:** 2018-06-30

**Authors:** Adrianna Potocka, Aleksandra Jacukowicz

**Affiliations:** 1 Nofer Institute of Occupational Medicine in Łódź, Department of Health and Work Psychology Łódź Poland

**Keywords:** nutritional status, diet, preschool children, geographic factors, region of residence, stan odżywienia, dieta, przedszkolaki, czynniki geograficzne, region zamieszkania

## Abstract

**Introduction and aim:**

Proper nutritional status in early childhood makes it possible for children to achieve their genetically-determined growth potential and intelligence. A child’s nutritional status is due to economic, cultural, psychosocial and geographic factors. The present study aims to check whether the nutritional status of preschoolers differs depending on their place of residence.

**Materials and methods:**

In order to achieve this goal we used 24-h dietary recalls to assess the children’s diet based on interviews with 530 mothers of preschool children from five different regions of Poland. Moreover, the children’s BMIs were calculated.

**Results:**

We found differences in the level of anthropometric indicators, diet structure and what percentage of the estimated average requirement for energy was met depending on the region where the children live. Youngsters from the western region of Poland had significantly higher BMIs than those from other regions. What is more, the percentage of meeting the estimated average requirement for energy and the percentage of calories from carbohydrates was higher among children from the western region, as compared with preschoolers from other areas of the country. The amount of fats and protein in the diet of children from western Poland, in turn, was significantly lower as compared with the diet of their peers from other regions.

**Conclusions:**

The present study provides evidence that the nutritional status of children should be analyzed in the context of their place of residence. Thus, the differences between regions and their impact on the health status of their inhabitants should be taken into account when designing preventive or corrective measures.

## Streszczenie

**Wprowadzenie i cel:**

Właściwy stan odżywienia we wczesnym okresie życia pozwala osiągnąć genetycznie uwarunkowany potencjał wzrostowy oraz poziom inteligencji. Na stan odżywienia dziecka wpływ mają czynniki ekonomiczne, kulturowe, psychospołeczne oraz geograficzne. Celem badania było ustalenie czy istnieją różnice w stanie odżywienia dzieci ze względu na ich miejsce zamieszkania.

**Materiał i metody:**

Badaniem objęta została grupa 530 matek dzieci w wieku przedszkolnym zamieszkująca pięć regionów Polski. Aby ocenić strukturę diety dzieci, przeprowadzono z matkami 24 godzinny wywiad żywieniowy. Ponadto oszacowano wskaźniki BMI.

**Wyniki:**

Zaobserwowano istotnie różnice w wartościach wskaźnika BMI dzieci oraz w strukturze i wartości energetycznej ich diety ze względu na miejsce zamieszkania. Dzieci z regionu zachodniego miały istotnie wyższe wskaźniki BMI w porównaniu do dzieci zamieszkujących inne regiony. Ponadto dieta dzieci z regionu zachodniego, w porównaniu do diety dzieci z pozostałych regionów, charakteryzowała się istotne większym procentem realizacji normy na energię i procentem energii pochodzącej z węglowodanów. Z kolei procent energii pochodzącej z tłuszczy oraz białka w diecie dzieci matek zamieszkujących region zachodni były istotnie niższe w porównaniu do pozostałych regionów. Dieta dzieci matek z regionu zachodniego, w porównaniu do diety dzieci matek z pozostałych regionów, charakteryzuje się również istotne większym procentem realizacji normy na energię.

**Wnioski:**

Wyniki badania pokazują, że stan odżywienia dzieci powinien być analizowany w kontekście ich miejsca zamieszkania. Tworząc zdrowotne programy profilaktyczne warto mieć na uwadze, co różni poszczególne regiony i czy różnice te mogą mieć znaczenie dla kształtowania stanu zdrowia ich mieszkańców.

## Introduction

Childhood obesity already affects more than one in four school-age children in Europe and in many countries the prevalence is increasing [1]. The number of young children (aged 5 or less) with excessive weight is also rapidly growing [2]. The problem of malnutrition also refers to Poland − 1/3 of all school-age children suffer from overweight or obesity [3]. Yet, even though previous studies suggest that malnutrition occurs in Polish children of all age groups, it is hard to estimate the exact scale of the problem in younger children (preschoolers), since research on the nutritional status involving this age group is particularly scarce in Poland [4].

The nutritional status of children might differ depending on their place of residence, as revealed by some research findings [5, 6, 7]. The differences in the nutritional status of children living in various regions of Poland, as well as different places in other countries, might be due to socioeconomic (poorer versus wealthier regions) and cultural factors (eating habits and traditions, regional cuisine). Some authors argue that programs addressing childhood obesity that consider the role of the particular region’s specificity are more effective [8, 9, 10, 11]. The differences in people’s attitudes and habits make some preventive and corrective actions more or less efficient [8]. For instance, in France the promotion of healthy eating proved more effective when the focus of communication was shifted onto the pleasure related to healthy eating and working out, without focusing only on the health benefits themselves [12].

To prevent malnutrition also among Polish children, it is necessary to conduct more research in this field, especially to run studies on preschoolers including their place of residence as a potential factor. Thus, in the present study we aimed to check whether the indices of nutritional status vary between the children living in different areas of the country. To the best of our knowledge, this is the first study including a national sample of Polish mothers that investigates the place of residence as a factor differentiating the preschoolers’ nutritional status. The present study constitutes part of a larger research project on the sociodemographic determinants and the role of the mother in the development of malnutrition in preschool children. The publication was prepared within the framework of a statutory project of the Nofer Institute of Occupational Medicine (IMP 21.3).

## Material and methods

### Procedure and participants

The study sample included 530 mothers of preschool children from five regions of Poland – the central, western, eastern, northern and southern one, each represented by one voivodeship. The central region was represented by the Masovian Voivodeship, the western one by the Lubusz Voivodeship, the eastern region by the Lublin Voivodeship, the northern one by the Pomeranian Voivodeship and the southern region by the Silesian Voivodeship. Two pediatric clinics were randomly selected from each region – one located in an urban and the other in a rural municipality. From these randomly selected institutions we recruited mothers who had at least one child aged 3-5 years. To avoid the confounding effect of any third parties engaged in the child’s diet, we excluded mothers who self-assessed their control over the child’s diet as lower than 70% and those whose children were being taken care of by babysitters (>2 hours a day) or grandparents living in the same household. The statistical analyses were performed on a sample of 400 women − 80 respondents from each region (40 from urban and 40 from rural areas). The mothers were on average 30.4 years old (+/-4.4). Women with secondary (N=172) and bachelor-degree education (N=93) dominated in the sample (43% and 24%, respectively). Vocational education was declared by 19% (N=74) of the respondents, a Master’s degree by 13% (N=51) and primary education by 3% (N=10) of the women.

### Measures

To gather the data, trained interviewers conducted face-to-face, structured interviews with the mothers. The questionnaires included:

– the socio-demographic characteristics of the mothers and children − place of residence (region of Poland), age, personal and occupational status
– the child’s nutritional status:
  - Body Mass Index (BMI) z-score for age [13], based on the data declared by the mothers;
  - the chils's diet structure: information on the amount and type of food consumed by the child during the day preceding the study based on a 24h-dietary recall filled in by the mother. Standard portion sizes were estimated using the food photographs album developed by the National Food and Nutrition Institute [14]. Based on this information, we calculated: the percentage of meeting the estimated average requirement for energy (EAR %) and the percentage of energy in the child’s diet coming from carbohydrates (EC %), fat (EF %) and proteins (EP %).

### Statistical analysis

To assess the diet structure, Dieta5 software was used [15]. We used one-way ANOVA to compare the indices of the children’s nutritional status and diet structure. All the statistical analyses were performed using STATISTICA 10.

## Results

The ANOVA results showed significant differences as regards all the analyzed indices of nutritional status (BMI, EAR %, EC %, EF % as well as EP %) among children from different regions of Poland (Table I).

**Table I:** Differences in the indices of children’s nutritional status depending on their place of residence(N=400) Tablela I. Różnice we wskaźnikach stanu odzywienia dzieci w zależności od miejsca zamieszkania.

Region *Region*	Northern *Północny*		Central *Centralny*		Western *Zachodni*		Eastern *Wschodni*		Southern *Południowy*		F
	M	SD	M	SD	M	SD	M	SD	M	SD	
BMI	14.80	1.40	14.84	1.74	18.32	2.70	14.65	1.45	14.37	1.16	68.84***
%EAR	80.10	27.17	85.05	26.34	95.31	40.81	74.92	22.10	89.88	19.32	6.46***
%EC	55.43	7.73	55.56	7.23	60.57	8.13	54.02	7.60	55.61	6.91	8.89***
%EF	30.21	7.63	29.36	7.37	26.05	8.09	30.67	7.35	29.36	6.46	4.80**
%EP	14.36	3.58	15.06	2.63	13.38	3.18	15.31	3.67	15.02	3.30	4.48**

*Note*: BMI – Body Mass Index/*wskaźnik masy ciała*, EAR − Estimated Average Requirement/*średnie zapotrzebowanie na energię*, EC – energy from carbohydrates/*energia z węglowodanów*, EF – energy from fat/*energia z tłuszczu*, EP – energy from proteins/*energia z białka* **p<0.01; ***p<0.001.

**Fig. 1:**
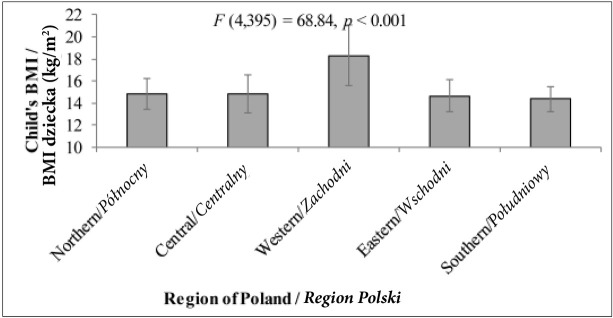
A child’s BMI depending on the place of residence (mean+/-standard deviation) Ryc. 1. BMI dziecka w zależności od miejsca zamieszkania (średnia +/- odchylenie standardowe).

**Fig. 2:**
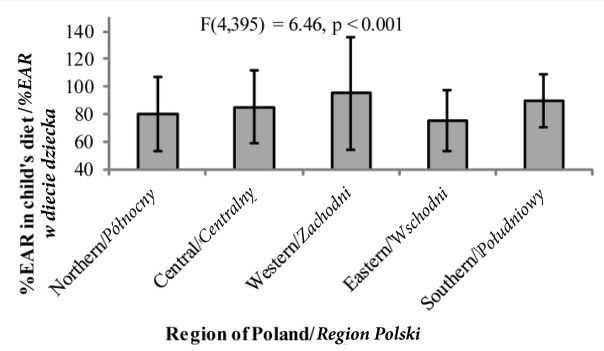
The percentage of the realization of the norm for energy intake − estimated average requirement (%EAR) − depending on the place of residence (mean+/-standard deviation) Ryc. 2. Procent realizacji normy na energię − średnie zapotrzebowanie dla grupy (%EAR) − w zależności od miejsca zamieszkania (średnia +/- odchylenie standardowe).

**Fig. 3:**
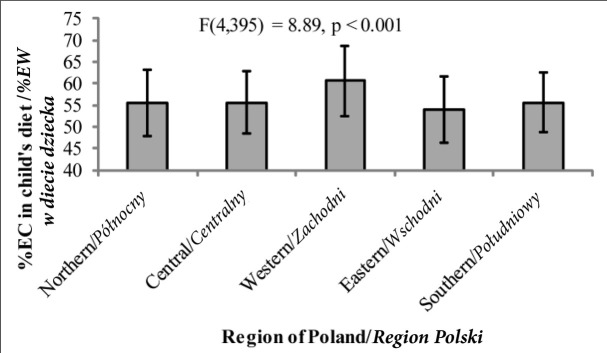
The percentage of energy in a child’s diet coming from carbohydrates (%EC) depending on the place of residence (mean +/- standard deviation) Ryc. 3. Procent energii w diecie dziecka pochodzący z węglowodanów (%EW) w zależności od miejsca zamieszkania (średnia +/- odchylenie standardowe).

**Fig. 4:**
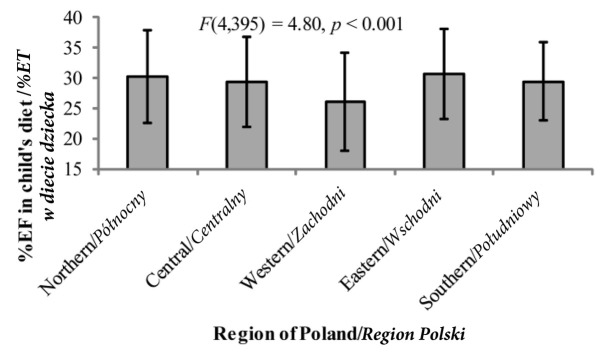
The percentage of the energy in child’s diet coming from fat (%EF) depending on the place of residence (mean +/- standard deviation). Ryc. 4. Procent energii w diecie dziecka pochodzący z tłuszczy (%ET) w zależności od miejsca zamieszkania (średnia +/-odchylenie standardowe).

**Fig. 5:**
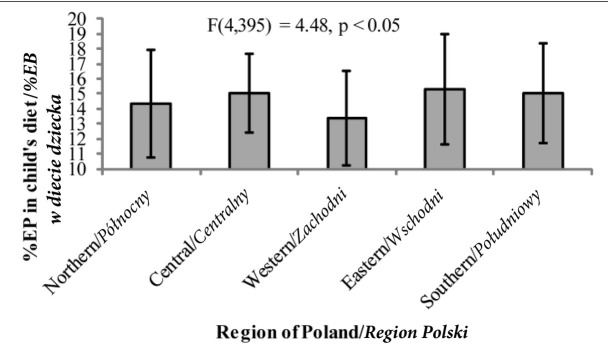
The percentage of energy in a child’s diet coming from proteins (%EP) depending on the place of residence (mean +/- standard deviation). Ryc. 5. Procent energii w diecie dziecka pochodzący z białka (%EB) w zależności od miejsca zamieszkania (średnia +/-odchylenie standardowe).

*Post hoc* analyses (the Bonferroni test) revealed that children from western Poland achieved significantly higher scores as regards BMI than children from all the other regions (Figure 1). Also %EAR (Figure 2) among children from the western region was significantly higher than that of children from northern and eastern Poland (the Bonferroni *post hoc* test significant at *p* < 0.01 in both cases). Additionally, children from the eastern and southern regions differed as regards their %EAR (the Bonferroni **post** *hoc* test significant at *p*<0.01).Similarly, %EC (Figure 3) in children from the western regions was also significantly higher and %EF significantly lower (Figure 4) than those of children from the other regions of Poland (the Bonferroni *post hoc* test significant at *p*<0.05 in all cases). Also %EP among children from western Poland was significantly lower than that of children from the central, eastern and southern region of the country (the Bonferroni test – *p* < 0.05) (Figure 5).

## Discussion

The results showed significant differences in the nutritional status of children depending on their place of residence. Such findings confirm that the place of residence influences the differences in the foods consumed and thus the nutritional status of the person [5, 16, 17, 18]. Our results also showed that the structure of the children’s diet was different in western Poland, as compared to other regions. The level of meeting the norm for energy intake (%EAR) was higher among children from the west as compared to children from other regions. Moreover, the percentage of energy coming from carbohydrates (%EC) in the diet of children from the west (60.6%) was higher than that of children from other regions and exceeded the recommended level of 50-55% [19]. Hence, it is probable that higher BMI values among children from the western region resulted from high %EAR and the excessive intake of carbohydrates. Further, the percentage of energy coming from other sources − fat and proteins − was significantly lower in the diet of children from the western region in comparison to other children.

Our results revealed that the indices of the nutritional status of children from western Poland turned out to be highly distinctive in comparison to children from other regions. First, the variance analysis revealed that the average BMIs of children living in the west were significantly higher than those of children from the other regions. Such a result implies that children from western Poland are at a higher risk of overfeeding disorders in comparison to other children. To some extent, such results correspond to those of the National Food and Nutrition Institute [5] which showed that excessive body mass among 9-year-olds was most common among children from central and western Poland, and least common in the south. Moreover, a Polish nationwide study revealed differences in the basic anthropological parameters between children from various regions of the country [6]. The children and teenagers who were studied (aged 7-18) and came from eastern Poland had lower height and weight in comparison to the respondents from other regions. Similarly, our results showed that children from the east achieved the second lowest BMIs in the sample (the lowest were observed among children from the south). Nevertheless, the studies by the National Food and Nutrition Institute [5], as well as by Kułaga and colleagues, [6] involved older children than those in our sample, thus the possibility for comparison is somewhat limited.

In general, we observed the most contrasting results as regards the diet structure between the children from the western and eastern regions of Poland. The children from the west had a higher total calorie intake, their diets were higher in carbohydrates and lower in fat and proteins in comparison to the children from the east. Hence, the comparison of these two regions points to the conclusion that the children from the west ate a carbohydrate diet, whereas the diet of children from the east was higher in proteins and fats. Such discrepancies could be hypothetically interpreted as a result of the differences in regional cuisines. However, the everyday diet in eastern Poland is richer in carbohydrates (high consumption of pierogi – dumplings, porridge and potato dishes, typical of Kresy – i.e. the Eastern Borderlands) than in proteins (meat is considered more as a side, rather than a main dish) [20]. The diet in the western region, in turn, is more eclectic, combining dishes characteristic for the Eastern Borderlands, Greater Poland, Germany and many others. Therefore, there should be no considerable differences between the ordinary cooking in these two regions. Moreover, it can be assumed that economic factors can influence the typical food consumption. Low-income families eat more foods rich in carbohydrates, such as bread, potatoes and sugar, whereas high-income families are reported to consume more beef, fish, cheese and yoghurts, i.e. products high in proteins [21]. Data on the financial situation of Polish households for 2012 demonstrate that in the Lublin Voivodeship (eastern Poland) 35% of the families were classified as the lowest-income group, whereas in the Lubusz Voivodeship (western Poland) there were only 21% of such families. There were no clear differences between these regions as regards the number of households classified as having the highest income [22]. Hence, it could be assumed that the diet of children from the eastern region would be richer in carbohydrates, rather than in proteins, yet our results showed the opposite results. Therefore, we must consider the possibility that these differences are not due to the local, regional cooking or economic factors and it is not these that have a major impact on the nutritional status of children but the people’s eating habits, such as snacking or adding sugar to beverages. It is probable that children from the western regions eat more sweets or white sugar, hence the higher consumption of carbohydrates and BMI. The relationship between weight excess and an improper diet structure has already been investigated by Goluch-Koniuszy and colleagues [23] who assessed the diet of 13-year-olds living in northwestern Poland. The authors found that overweight and obesity in children could be caused by excessive consumption of simple sugar coming from sweets, sweet baked products, sweetened soft and energy drinks. Our results, along with those by Goluch-Koniuszy and colleagues [23], indicate the need for comprehensive eating assessment including an in-depth qualitative analysis focused not only on the amount of the food consumed, but mainly on the kind of foods eaten by children. Such an assessment should also involve the analysis of eating habits, which are shaped by social (culture, eating habits and traditions prevalent in the given region), as well as individual factors (attitudes, eating preferences, mental status, succumbing to external influence). Only a detailed and comprehensive analysis of local, regional, country-specific factors along with individual ones makes it possible to develop effective preventive programs addressed to the nutritional status of Polish preschoolers.

## Conclusions

The present study provides evidence that the nutritional status of children should be analyzed in the context of their place of residence. Thus, the differences between regions and their impact on the health status of their inhabitants should all be taken into account when designing preventive or corrective measures.
